# In Situ Growth of Zeolitic Imidazolate Framework-L in Macroporous PVA/CMC/PEG Composite Hydrogels with Synergistic Antibacterial and Rapid Hemostatic Functions for Wound Dressing

**DOI:** 10.3390/gels8050279

**Published:** 2022-05-01

**Authors:** Hang Yang, Xianyu Lan, Yuzhu Xiong

**Affiliations:** College of Materials and Metallurgy, Guizhou University, Guiyang 550025, China; gs.mjsong19@gzu.edu.cn (H.Y.); gs.lanxy21@gzu.edu.cn (X.L.)

**Keywords:** in situ growth, macroporous structure, synergistic antibacterial, rapid hemostatic, carboxymethyl cellulose, wound dressing, hydrogel, ZIF-L, polyvinyl alcohol, phase separation technique

## Abstract

Although many advances have been made in medicine, traumatic bleeding and wound infection are two of the most serious threats to human health. To achieve rapid hemostasis and prevent infection by pathogenic microbes, the development of new hemostatic and antibacterial materials has recently gained significant attention. In this paper, safe, non-toxic, and biocompatible polyvinyl alcohol (PVA); carboxymethyl cellulose (CMC), which contains several carboxyl and hydroxyl groups; and polyethylene glycol (PEG), which functions as a pore-forming agent, were used to prepare a novel PVA/CMC/PEG-based composite hydrogel with a macroporous structure by the freeze-thaw method and the phase separation technique. In addition, a PVA/CMC/PEG@ZIF-L composite hydrogel was prepared by the in situ growth of zeolitic imidazolate framework-L (ZIF-L). ZIF-L grown in situ on hydrogels released Zn^2+^ and imidazolyl groups. They elicited a synergistic antibacterial effect in hemostasis with PVA and CMC, rendering the PVA/CMC/PEG@ZIF-L hydrogel with a good antibacterial effect against *Staphylococcus aureus*. At the same time, the macroporous structure enabled the rapid release of Zn^2+^ and imidazolyl groups in ZIF-L and promoted cell proliferation at an early stage, enhancing the coagulation efficiency. A rat liver injury model was used to confirm its rapid hemostasis capacity.

## 1. Introduction

Traumatic bleeding is one of the main causes of civilian and military deaths [1]. According to a previous report, more than 5.8 million deaths each year are caused by excessive bloodshed worldwide [2]. As such, the rapid control of bleeding is the key to reducing mortality. However, secondary tissue damage caused by bacterial infections is a major problem associated with the rapid control of bleeding [3,4,5]. Although cotton gauzes, zeolite-based Quik Clot, and other hemostatic products are commercially available, they show hemostatic effects and possess different shortcomings in that they do not meet the requirements for antibacterial control, which limits their wide clinical application [6]. Therefore, it is of great significance to develop safe and rapid hemostatic agents or wound dressings with an excellent antibacterial ability to treat severe bleeding and subsequent trauma [7].

Many investigators continue to conduct important research on hemostatic and antibacterial hydrogel wound dressings. According to the hydrogel mechanism of action and the antibacterial mechanism of action, the currently available antibacterial hydrogels can be divided into three categories; namely, hydrogels with antibacterial activity, hydrogels loaded with antibacterial agents, and stimulus-responsive hydrogels [8,9,10,11]. Chitosan, gelatin, cellulose, polyols, and alginates are the main representatives of their own antibacterial active substances. There are single hydrogels that use the antibacterial substance as the matrix as well as composite hydrogels such as alginate-based hydrogels [12,13], chitosan-based hydrogels [14], and PVA/chitosan-based hydrogels [15]. Amongst them, polyvinyl alcohol (PVA) hydrogels can be formed by physical cross-linking through safe, non-toxic, and simple freeze-thaw means; it is biodegradable, non-toxic, biocompatible, and hydrophilic [16]. These characteristics are favored by researchers. Hydrogels loaded with antibacterial agents have recently received much attention. Antibiotics (e.g., vancomycin hydrochloride [17] and gentamicin [18]) have successfully been loaded onto hydrogels [12,13,19]; the antibacterial agents generally are metal ions (e.g., Ag^+^, Zn^2+^, and Cu^2+^ [20]), metal–organic framework (MOF) materials [21], antibacterial polypeptides [22,23,24], and biological extracts [25,26]. A variety of antibacterial hydrogels with a controlled release of metal ions can be prepared through metal coordination bonds between metal ions and polymer matrices such as Zn^2+^ and Sr^2+^ double-ion cross-linked hydrogel membranes [27] and Ag^+^-loaded hydrogels [28]. At the same time, the introduction of metal coordination bonds can enhance the mechanical strength of hydrogels [29]. Other studies have reported that zeolite-like imidazole ester framework materials (ZIFs) have excellent antibacterial properties. For example, air filters that are combined with ZIF-8 particles exhibit an excellent inactivation efficiency against airborne bacteria [30]. ZIFs can not only slowly release Zn^2+^ during wound healing and effectively kill bacteria by destroying microbial cell membranes, but also accelerate wound healing by promoting cell migration, angiogenesis, and collagen deposition [31]. There are many studies on final stimulus-responsive antibacterial hydrogels in the field of wound healing [32,33,34,35,36]. Although several hemostatic and antibacterial hydrogels have been developed, many hydrogels are not applicable to the rapid control of bleeding and further research is needed.

According to the ideal state of wound dressing [37] and the theory of wet wound healing [38], this paper selected safe, non-toxic, and biocompatible PVA as well as carboxymethyl cellulose (CMC) with several carboxyl and hydroxyl groups as the hydrogel matrices. Both PVA and CMC not only have certain antibacterial properties, but the carboxyl and hydroxyl groups in CMC can also chelate with zinc ions. A zeolitic imidazolate framework-L(ZIF-L) was then formed in situ in the hydrogel by combining Zn^2+^ with the imidazolyl groups. ZIF-L has antibacterial properties and provides rapid hemostasis in hydrogels [39,40]. Polyethylene glycol (PEG) was used as the pore-former of the gel. The hydrogel was prepared by phase separation using PEG to create its macroporous structure. In the prepared PVA/CMC/PEG@ZIF-L composite hydrogel, the Zn^2+^ and imidazolyl groups released by ZIF-L, PVA, and CMC exhibited synergistic antibacterial effects in hemostasis, indicating that the PVA/CMC/PEG@ZIF-L composite hydrogel had a good antibacterial effect. At the same time, the macroporous structure of the hydrogel not only provided the hydrogel with a good gas exchange capacity and the rapid absorption of blood to allow platelets and blood cells to aggregate to cause blood coagulation, but also allowed the Zn^2+^ and imidazolyl groups in ZIF-L to be rapidly released, allowing the hydrogel to quickly control bleeding.

## 2. Results and Discussion

### 2.1. Structure Analysis

**Figure 1:**
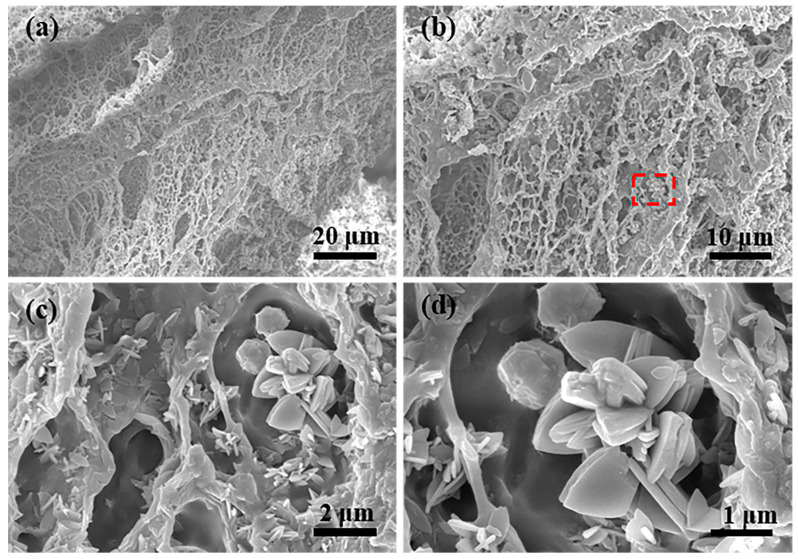
SEM images of the PVA/CMC/PEG@ZIF-L hydrogel: (**a**) magnified 1000 times, scale bar is 20 μm; (**b**) magnified 2000 times, scale bar is 20 μm; (**c**) magnified 10,000 times, scale bar is 2 μm; (**d**) magnified 30,000 times, scale bar is 1 μm.

**Figure 2:**
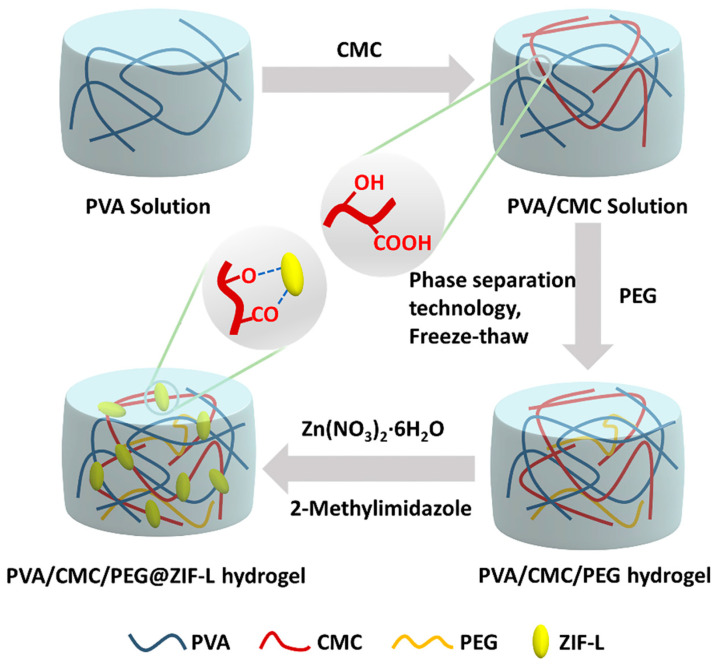
The formation of the PVA/CM/PEG@ZIF-L hydrogel and the in situ growth process of ZIF-L on the hydrogel network.

The cross-sectional SEM images of the PVA/CMC/PEG@ZIF-L composite hydrogel are shown in Figure 1a–d. The hydrogel had a three-dimensional, network-like, and porous structure, and the average pore size of the PVA/CMC/PEG@ZIF-L composite hydrogel was 1.76 μm, which was close to 2 μm. The standard deviation was 0.6436 μm. It indicated that PEG fully exerted its role as a pore-forming agent. A macroporous structure was prepared by phase separation. The pore wall of the pure PVA/CMC/PEG composite hydrogel without ZIF-L was relatively smooth whereas the surface of the PVA/CMC/PEG@ZIF-L composite hydrogel was rough, with leaf-like crystals observed on the gel network. The leaf-like crystals were ZIF-L [41,42]. There were many ZIF-L nanosheets in the pore wall of the hydrogel, indicating that ZIF-L was successfully formed in situ in the PVA/CMC/PEG hydrogel. The in situ growth process of ZIF-L on the hydrogel network is illustrated in Figure 2. When the PVA/CMC/PEG hydrogel was immersed in the Zn (NO_3_)_2_·6H_2_O aqueous solution, a large amount of Zn^2+^ was adsorbed by the carboxymethyl and hydroxyl groups in the hydrogel and attached to the surface and pore walls of the hydrogel. It was soaked in a 2-methylimidazole aqueous solution; the imidazole group and Zn^2+^ were combined and ZIF-L grew in situ on the hydrogel network.

**Figure 3:**
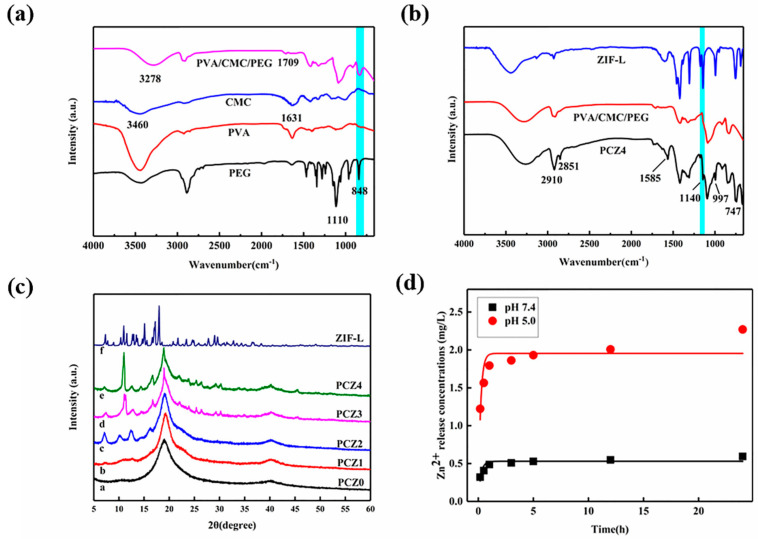
(**a**) Fourier infrared spectra of PVA, CMC, PEG, and PVA/CMC/PEG hydrogels; (**b**) Fourier infrared spectra of PVA/CMC/PEG, ZIF-L, and PCZ4 hydrogels; (**c**) XRD spectra of ZIF-L and PCZ with different amounts of ZIF-L; (**d**) fitted curve of the first−order kinetic model of the Zn^2+^ release of the PCZ4 hydrogel at different pH values.

Figure 3a is the infrared spectrum of the PVA/CMC/PEG hydrogel. The absorption peaks at 848 cm^−1^ and 1090 cm^−1^ corresponded with the characteristic peaks of the ether bond (-O-) in PEG [43], and the corresponding characteristic peaks were also clearly observed in the PVA/CMC/PEG composite hydrogel. This indicated that PEG was entangled with the PVA molecular chain during the freezing-thawing process and not completely precipitated, which was subsequently condensed into water. The mechanical properties of the glue played a certain enhancement role. In addition, 1631 cm^−1^ in the CMC spectrum corresponded with the stretching vibration peak of COO- in the carboxymethyl group of CMC [44] and 3460 cm^−1^ corresponded with the stretching vibration peak of -OH of CMC. In the PVA/CMC/PEG hydrogel spectrum, the stretching vibration peaks of COO- and -OH corresponded with 1709 cm^−1^ and 3278 cm^−1^, respectively. In the PVA/CMC/PEG hydrogel spectrum, the stretching vibration peak of -OH at 3278 cm^−1^ broadened, indicating hydrogen bonding between the hydroxyl group of PVA and the carboxymethyl group of CMC [45]. Therefore, physical cross-links were formed between PVA and CMC during the freezing-thawing process, confirming the successful preparation of the PVA/CMC/PEG hydrogels. COO- of CMC was a broad peak; it was possible that COO- also formed intermolecular hydrogen bonds. Furthermore, COO- shifted in the PVA/CMC/PEG hydrogel spectrum, which might be the result of the interaction between PVA and CMC [45]. Although the stretching vibration peaks of COO- and -OH shifted in the PVA/CMC/PEG hydrogel spectrum, these findings indicated that COO- and -OH existed in the PVA/CMC/PEG hydrogel, providing a basis for the chelation of carboxyl, hydroxyl, and Zn^2+^.

The infrared spectrum of the PCZ4 hydrogel is shown in Figure 3b. The peak at 2910 cm^−1^ corresponded with the stretching vibration peak of methylene (-CH_2_-) of PCZ4 and the new peak at 2851 cm^−1^ was the water peak caused by the residual water. The peaks at 1585, 1140, 997, 747, and 423 cm^−1^ in the PCZ4 spectrum corresponded with the characteristic peaks of the ZIF-L crystal [46].

The XRD spectrum of ZIF-L and PCZ with different amounts of ZIF-L is shown in Figure 3c. The PVA/CMC/PEG hydrogel (PCZ0, which was without ZIF-L) had a strong diffraction peak at 2*θ* = 19.5°, which was similar to the (101) crystal in PVA. The diffraction peaks of ZIF-L at the (110), (200), (211), (220), (310), and (222) planes were observed at 2θ values of 7.30°, 10.95°, 12.71°, 15.11°, 17.2°, and 17.99°, respectively. This was similar to previous research [47,48,49,50]. Therefore, this confirmed that ZIF-L had been successfully synthesized. The sample containing ZIF-L (PCZ1, PCZ2, PCZ3, PCZ4) had a few new peaks, similar to the characteristic peaks of the ZIF-L crystal plane. These findings indicated that ZIF-L successfully grew in situ in the PVA/CMC/PEG hydrogel. In addition, the peak intensity in the PCZ sample increased with the increase in the ZIF-L crystal content. However, it might have been due to the influence of the PVA crystal planes on the ZIF-L crystal planes, resulting in a significant increase in the peak intensity only at 2*θ* ≅ 11°. Additionally, the lower the content of ZIF-L, the greater the influence of other substances (in this research, it was mainly reflected in the effect of PVA on ZIF-L) on its peak [51,52]. Hence, the characteristic peaks of ZIF-L in PCZ1 and PCZ2 were not significantly obvious and there was an apparent shift from 2*θ* ≅ 10° to 2*θ* ≅ 11° between PCZ2 and PCZ3.

### 2.2. Zinc Ion Release

The fitted curve of the first-order kinetic model of the Zn^2+^ release of the PCZ4 hydrogel at different pH values is shown in Figure 3d. During the first hour, the release rate of Zn^2+^ was fast, which was beneficial to the rapid hemostasis of the wound. Afterwards, the release rate of Zn^2+^ was relatively slow and stable, allowing it to act on the wound. The PVA, CMC, and imidazolyl groups produced by the hydrolysis of ZIF-L in the hydrogel also had certain antibacterial effects [11,12,13]. Zn^2+^ cooperated with other substances in the hydrogel for a synergistic antibacterial effect. Furthermore, the Zn^2+^ release of the PCZ4 hydrogel increased with the decrease in pH. ZIF-L in the PCZ4 hydrogel rapidly degraded under acidic conditions (pH 5.0) whereas the degradation was relatively slow under physiological conditions (pH 7.4) [34,53,54]. These results showed that the Zn^2+^ release from the PVA/CMC/PEG@ZIF-L composite hydrogel was pH-responsive and Zn^2+^ was easily released under acidic conditions. Human skin is finely acidic [55]; the pH responsiveness of the hydrogel was beneficial to hemostasis and antibacterial control. In addition, a first-order kinetic equation was fitted to the zinc ion release. Although the release of Zn^2+^ could be up to four-fold higher depending on the pH, the mechanism of the Zn^2+^ release was not affected as suggested by the model. The kinetic behavior of zinc ion release at different pH levels requires further research.

### 2.3. Biocompatibility Analysis

**Figure 4:**
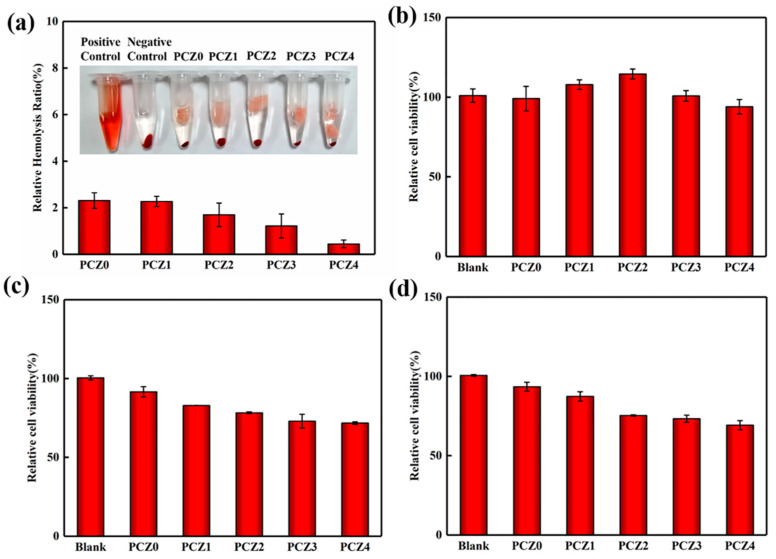
(**a**) Hemolysis rate of PCZ hydrogels with different amounts of ZIF-L. Relative cell viability of NIH-3T3 cells incubated for (**b**) 24 h; (**c**) 72 h; and (**d**) 120 h.

An ideal wound dressing requires a good compatibility with the vasculature and should not lyse red blood cells when it encounters blood. The blood compatibility of the dressing was evaluated by the hemolysis rate. The lower the hemolysis rate, the better the blood compatibility. The hemolysis rate of the PCZ hydrogel with different amounts of ZIF-L is shown in Figure 4a. The hemolysis rate of the PCZ hydrogel was less than 5%; as the amount of ZIF-L continued to increase, the hemolysis rate continued to decrease because ZIF-L generated in situ in the PVA/CMC/PEG composite hydrogel was superhydrophobic [56,57,58,59]. ZIF-L in the pores reduced the surface energy of the material, thereby reducing damage to the red blood cells [60]. Thus, the hemolysis rate was low. These results showed that the PVA/CMC/PEG@ZIF-L composite hydrogel had excellent blood compatibility and it met the criteria of wound dressings.

Cytotoxicity is another indicator of biocompatibility. After adding PCZ hydrogels with different amounts of ZIF-L to an NIH-3T3 cell suspension, the relative cell viability of the NIH-3T3 cells incubated for 24 h, 72 h, and 120 h was calculated and is shown in Figure 4b–d. In the hydrogels with different amounts of ZIF-L incubated for 24 h, we found that the relative cell viability of the NIH-3T3 cells was close to 100%. This showed that the PCZ hydrogels had excellent blood compatibility. In addition, PCZ1 and PCZ2 both promoted cell proliferation to varying degrees, which is conducive to wound healing. After 72 h of incubation, the relative cell viability in the hydrogel decreased and the relative cell viability of PCZ3 and PCZ4 was less than 80% because ZIF-L released Zn^2+^ and free 2-methylimidazole during hydrolysis. When Zn^2+^ in the leaching solution is too high, there is excessive generation of reactive oxygen species, which damages DNA. Furthermore, 2-methylimidazole is not conducive to cell survival. There was no difference in relative activity after 72 h and 120 h of incubation. The hydrolysis of ZIF-L slowed after 72 h, which was suggestive of complete hydrolysis. This had no effect on the cell survival. According to the biological evaluation standard of medical devices, the PVA/CMC/PEG@ZIF-L composite hydrogel met this standard and could be used as a wound dressing.

### 2.4. Hemostatic Analysis

The hemostatic efficiency of a wound dressing is related to its blood-clotting ability. A PVA/CMC/PEG@ZIF-L composite hydrogel can promote blood clotting based on two factors. On the one hand, because of its interconnected porous structure that can quickly absorb a large amount of blood, platelets and blood cells can aggregate to cause blood clotting. On the other hand, when a PVA/CMC/PEG@ZIF-L composite hydrogel contacts blood, ZIF-L gradually decomposes into Zn^2+^ and imidazolyl. Zn^2+^ can activate coagulation factors XII and VII to trigger the coagulation cascade and accelerate the production of thrombin and fibrin, thereby promoting coagulation [31]. ZIF-L may also slowly release Zn^2+^ through an exchange with Na^+^, activating the coagulation cascade. In addition, ZIF-L has a high porosity and large surface area, which can accelerate blood absorption and red blood cell/platelet coagulation. These two processes have a synergistic effect in the promotion of blood clotting.

**Figure 5:**
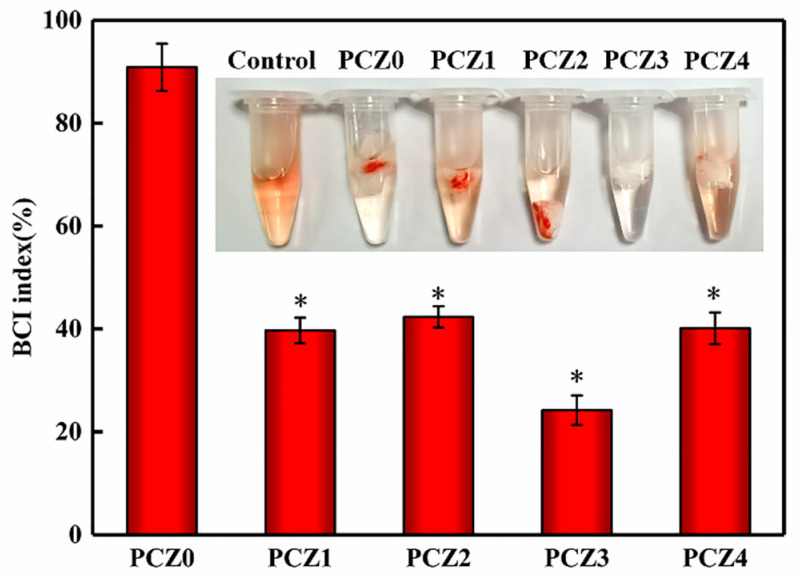
The coagulation index of PCZ hydrogels with different amounts of ZIF-L (* *p* < 0.05; *n* = 3).

Theoretically, the blood coagulation ability is inversely proportional to the coagulation index (BCI value). The coagulation index of the PVA/CMC/PEG@ZIF-L composite hydrogel with different amounts of ZIF-L is shown in Figure 5. The PVA/CMC/PEG hydrogel had a certain coagulation effect whereas the *BCI* value of the PVA/CMC/PEG@ZIF-L composite hydrogel was lower than that of the PVA/CMC/PEG hydrogel. PCZ1, PCZ2, PCZ3, and PCZ4 displayed coagulation effects with *BCI* values of 39.7 ± 2.5%, 42 ± 2.1%, 24.2 ± 2.9%, and 40.1 ± 3.1%, respectively. These findings indicated that ZIF-L played a role in promoting blood coagulation. In addition, as shown in Figure 5, it could be seen that the *BCI* value of PCZ3 was lower than PCZ4, indicating that the hemostasis efficiency of PCZ3 was higher than PCZ4. An excessive zinc ion concentration can produce too many reactive oxygen species (ROS) [30,31]. ROS not only cause oxidative damage to nucleic acid, but also damage proteins and biological cell membranes [61]. In the process of hemostasis, excess ROS might destroy blood cells, thrombin, and blood fibrin, which affect coagulation. Therefore, it was possible that the ZIF-L content of PCZ4 was too high, which produced too many reactive oxygen species and affected the hemostasis efficiency. In general, the PVA/CMC/PEG@ZIF-L composite hydrogel could promote blood coagulation.

**Figure 6:**
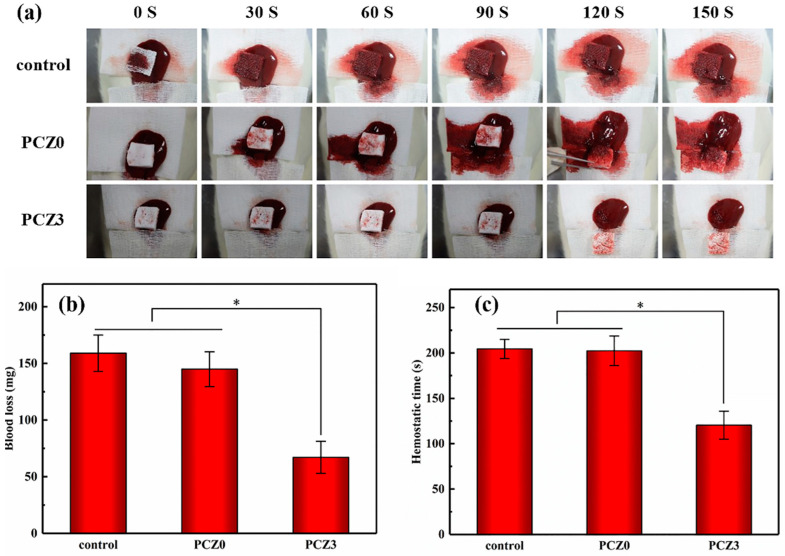
In vivo evaluation of the hemostatic capacity of different hydrogels in a rat liver trauma model. (**a**) Macroscopic images; (**b**) blood loss; and (**c**) time to hemostasis in a hemostatic application (* *p* < 0.05; *n* = 3).

The coagulation properties of the samples were further assessed using a rat liver injury model by recording the time to hemostasis and the amount of blood loss leading to hemostasis (Figure 6). As shown in Figure 6b, the amount of blood loss (67 mg) caused by PCZ3 was compared with a medical gauze and PCZ0 without ZIF-L. The hemostatic time followed the same trend in the overall loss of blood. In Figure 6c, it can be seen that the hemostasis time of the medical gauze, PCZ0, and PCZ3 was 204 s, 202 s, and 120 s, respectively. PCZ3 had the shortest hemostatic time. The coagulation ratio was consistent with the image after a hemorrhag (Figure 6c). The results showed that the PVA/CMC/PEG @ZIF-L hydrogel had a fast hemostatic ability.

### 2.5. In Vitro Antibacterial Assays

**Figure 7:**
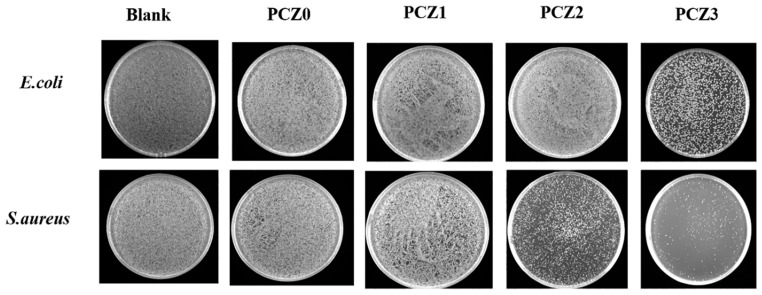
In vitro antibacterial colonies of PCZ hydrogels with different amounts of ZIF-L.

The in vitro antibacterial effects of the PCZ hydrogel with different amounts of ZIF-L are shown in Figure 7. As the content of ZIF-L increased, the content of released zinc ions also increased and the antibacterial effect of the hydrogel gradually improved, especially for *Staphylococcus aureus*. ZIF-L has inherent antibacterial properties [39,40] due to the release of Zn^2+^ and imidazole groups. The imidazole groups can destroy the liposome structure of bacteria. In addition, Zn^2+^ can interact with the cell membrane of bacteria and penetrate the cell wall to destroy its protein and DNA, leading to cell death [31,39,40]. Compared with *Escherichia coli*, the hydrogel had a better antibacterial effect against *Staphylococcus aureus*. This might be because the main component of the cell wall of Gram-positive bacteria is peptidoglycan; peptidoglycan is located in the surface layer [62], so the antibacterial components in the hydrogel could act on it more quickly. As Gram-negative bacteria contain less peptidoglycan and are located in the inner layer [62], the antibacterial ingredients killed less *E. coli* than *Staphylococcus aureus* in the same time duration.

PVA and CMC also have certain antibacterial properties and they synergistically kill bacteria through the release of Zn^2+^ and imidazole groups by the hydrolysis of ZIF-L, thus giving PCZ excellent antibacterial effects against *Staphylococcus aureus*. Although the PVA/CMC/PEG@ZIF-L composite hydrogel had poor antibacterial effects against *E. coli*, requiring further research and improvement, its excellent antibacterial effects against *Staphylococcus aureus* could make the PVA/CMC/PEG@ZIF-L composite hydrogel a good wound dressing, exceeding many hemostatic products on the market.

## 3. Conclusions

Although several advancements have been made in medicine, traumatic bleeding and bacterial wound infections still pose a serious threat to human health. To solve this problem, many investigators have performed important research and developed new hemostatic and antibacterial materials, but most of these hydrogels still have shortcomings with respect to the rapid control of bleeding. In this paper, a PVA/CMC/PEG composite hydrogel was prepared using PVA and CMC as the raw materials and PEG as the pore-forming agent. The PVA/CMC/PEG@ZIF-L composite hydrogel was prepared by the in situ growth of ZIF-L on the hydrogel network. The morphologies and microstructures of the prepared hydrogels were characterized by SEM, FTIR, and XRD. The results showed that the PVA/CMC/PEG composite hydrogel and the PVA/CMC/PEG@ZIF-L composite hydrogel with a macroporous structure were successfully prepared by the freeze-thaw method and phase separation technology. In addition, to explore the biocompatibility of the PVA/CMC/PEG@ZIF-L composite hydrogel and the hemostatic and antibacterial mechanisms, a Zn^2+^ release test, blood compatibility test, cytotoxicity test, in vitro coagulation test, in vivo hemostasis test, and in vitro antibacterial test were carried out. The experimental results showed that the PVA/CMC/PEG@ZIF-L composite hydrogel was in line with the index of wound dressing and the various components in the PVA/CMC/PEG@ZIF-L composite hydrogel had synergistic antibacterial effects. As such, the gel had good antibacterial effects against *Staphylococcus aureus*. At the same time, the macroporous structure enabled the rapid release of Zn^2+^ and imidazolyl groups in ZIF-L and promoted cell proliferation at an early stage, thereby enhancing the coagulation efficiency.

## 4. Experimental Section

### 4.1. Materials

Polyvinyl alcohol (PVA1799, 99% degree of hydrolysis, 44.05 Mw, AR), carboxymethyl cellulose (CMC, USP), 2-methylimidazole (reagent purity: 98%), and methanol (AR) were purchased from the Aladdin Reagent Co., Ltd. (Shanghai, China). Polyethylene glycol (PEG2000, AR) and tert–butanol (AR) were purchased from the McLean Biochemical Technology Co., Ltd. (Shanghai, China). Zn (NO_3_)_2_·6H_2_O (AR) was purchased from the Comeo Chemical Reagent Co., Ltd. (Tianjin, China).

### 4.2. Preparation of PVA/CMC/PEG Composite Hydrogel

In brief, 2 g of PVA and 20 mL of deionized water were added to a blue cap reagent bottle. The PVA was dissolved in the deionized water and heated at 90 °C for 1 h. Thereafter, 0.2 g of CMC was added and stirred, followed by 1.5 g of PEG2000 with stirring and heating at 75 °C. After cooling to room temperature, the turbid liquid was poured into a mold (cylindrical PTFE, 3 cm diameter, about 2 cm height poured) and stored at −20 °C for 12 h. The frozen sample was carefully demolded and melted at room temperature to form the hydrogel. This was frozen and thawed once. The hydrogel was soaked in deionized water to remove PEG precipitated by the phase change to generate the PVA/CMC/PEG composite hydrogel. 

### 4.3. In Situ Growth of ZIF-L on PVA/CMC/PEG Composite Hydrogel

The PVA/CMC/PEG hydrogel was soaked in 0.119 g of Zn (NO_3_)_2_·6H_2_O in 30 mL of an aqueous solution for 24 h and then soaked in 0.263 g of 2-methylimidazole in 30 mL of an aqueous solution for 24 h to realize the in situ generation of ZIF-L in the hydrogel, which was denoted as PCZ1. The above experimental steps were repeated to achieve the soaking of the PVA/CMC/PEG composite hydrogel according to a two-fold concentration gradient of PCZ1, which was denoted as PCZ2. Likewise, PCZ3 followed a two-fold concentration gradient of PCZ2. The above steps were repeated for PCZ4. The resulting hydrogel was solvent-exchanged in a tert–butanol solution. To avoid the excessive shrinkage of the hydrogel, the content of tert–butanol in the solvent exchange process was gradually increased, followed by soaking in 50%, 75%, and 100% tert–butanol aqueous solutions for 1 h. The resulting hydrogel was then freeze-dried (vacuum degree 15 Pa, condensing temperature −50 °C, drying for 48 h) to obtain the product.

### 4.4. Testing and Characterization

#### 4.4.1. Scanning Electron Microscopy (SEM)

Scanning electron microscopy (SEM; SU8010, HITACHI Corporation, Tokyo, Japan) was used to observe the morphology of the PVA/CMC/PEG@ZIF-L composite hydrogel. The gel samples were freeze-dried and made brittle with liquid nitrogen; their microstructures were then observed by SEM. All samples had to be sprayed with gold before being tested. In the SEM images, 200 holes were selected for the pore size measurement. After the measurement and calculation, the average pore size was obtained.

#### 4.4.2. Fourier Transform Infrared Spectroscopy (FTIR)

Fourier transform infrared spectroscopy (FTIR; NEXUS6700, Thermo Company, Waltham, MA, USA) was used for the group characterization of the sample powder. KBr was added to 1–2 mg of sample solids and the samples were pressed and tested. The scanning beam range was 500–4000 cm^−1^. The gel samples were lyophilized and directly tested by ATR-FTIR.

#### 4.4.3. X-ray Diffraction (XRD)

X-ray diffraction (XRD; Empyrean PANalytical, Netherlands) was used to analyze the state of in situ growth of ZIF-L on the hydrogels with different ZIF-L contents. The characterization of hydrogels was undertaken after freeze-drying. The scanning speed was 0.2 s/step and the scanning angle range was 5~80°.

#### 4.4.4. Zinc Ion Release

Inductively coupled plasma spectrometry was used to determine the release of Zn^2+^ from the PVA/CMC/PEG@ZIF-L composite hydrogel. A total of 0.2 g of PCZ4 hydrogel was dipped into 30 mL of PBS at a pH of 5.0 and a pH of 7.4 and the stirring speed was adjusted to 100 rpm. According to the crystallization output method, the solubility of ZIF-L in PBS with a pH of 5 and with a pH of 7.4 was 30 mg/100 g and 6 mg/100 g, respectively. After 10 and 30 min as well as 1, 3, 5, 12, and 24 h, a 2 mL aliquot was used to determine the Zn^2+^ release. After removing each 2 mL aliquot, 2 mL of PBS was added to ensure that the hydrogel soaking solution was always 30 mL.

#### 4.4.5. Biocompatibility Test

The biocompatibility test was divided into the blood compatibility test and the cytotoxicity test. For the blood compatibility test, 4.0 mL of peripheral blood was drawn from a healthy volunteer using a vacuum blood collection tube for a total of two tubes with 3.2% sodium citrate anticoagulation. After a tube of anticoagulated whole blood was centrifuged for 10 min, 0.4 mL of a red blood cell pellet was drawn into a 1.5 mL centrifuge tube. A total of 1.0 mL of normal saline was then added to the centrifuge tube and mixed with the red blood cells, centrifuged for 10 min, and the supernatant was aspirated. Subsequently, 0.5 mL of physiological saline was added to the centrifuge tube to dilute the red blood cells to obtain a red blood cell suspension. After obtaining the red blood cell suspension, 10 mg of the sample was placed in a 1.5 mL centrifuge tube, 1 mL of physiological saline was added, and then 20 μL of the red blood cell suspension was added, followed by shaking at 37 °C for 4 h, centrifugation for 5 min, and the aspiration of the supernatant. A microplate reader was used to measure the absorbance at 545 nm. Thereafter, 20 mL of the erythrocyte suspension was added to 1 mL of physiological saline as a negative control and 20 mL of the erythrocyte suspension was added to 1 mL of tri-distilled water as a positive control. The centrifuge tubes were imaged. The relative hemolysis rate of the sample was calculated according to the following formula:

(1) $$\mathrm{Hemolysis Rate}\;(\%)=\frac{D_{t}-D_{nc}}{D_{pc}-D_{nc}}\times 100\%$$

where $D_{t}$, $D_{nc}$, and $D_{pc}$ are the adsorptions, negative control, and positive control of samples, respectively. A relative hemolysis rate > 5% was regarded as a hemolysis phenomenon.

The in vitro cytotoxicity test used the MTT method. The ratio of calf serum:sodium pyruvate:glutamine:non-essential amino acid was 87:10:1:1 and a DMCM high-glucose medium was prepared. The NIH-3T3 cells were cultured at 4 × 10^3^/well in 96-well plates for 24 h at 37 °C. The samples were cut into small pieces after sterilization by UV light for 30 min; 100 mg of the samples was added to 1 mL of a DMEM high-glucose medium and placed in a 37 °C, 5% CO_2_ incubator for 24 h. After centrifugation, the supernatant was taken and filtered with a 0.22 μm membrane to obtain a sample extract with a concentration of 100 mg/mL. The leaching solution was diluted to 10%, 20%, 50%, 80%, and 100% and added to a 96-well plate at 100 μL/well, respectively. The control group was the DMEM medium of the NIH-3T3 cells without an extracting solution. Three replicate wells were made for each treatment and cultured for 24 h, 72 h, and 120 h, respectively. The medium containing the samples was then removed, each well was washed 3 times with PBS, and 100 μL of an MTT medium containing 0.5 mg/mL was added to each well. This was incubated in a 5% CO_2_, 37 °C constant temperature incubator for 4 h and 100 μL of DMSO was added to each well of the supernatant. Finally, it was shaken for 10 min to completely dissolve the crystal and the absorbance at 570 nm was detected. The absorbances of the experimental group and the control group at 570 nm were recorded as *OD_E_* and *OD_C_*, respectively. The following is the formula used to calculate the relative viability of cells:

(2) $$\mathrm{Relative Viability}\;(\%)=\frac{OD_{E}-\mathrm{Background values}}{\sum OD_{c}/n-\mathrm{Background values}}\times 100\%$$

The experimental group used a hydrogel extract (100 mg/mL) with different amounts of ZIF-L and the control group did not contain a hydrogel extract.

#### 4.4.6. In Vitro Coagulation Test

The coagulation index *BCI* (blood-clotting index) was used for the in vitro coagulation test. PCZ0, PCZ1, PCZ2, PCZ3, and PCZ4 were cut and 10 mg was placed in a 1.5 mL centrifuge tube and kept at 37 °C for 5 min. A total of 5 μL of recalcified anticoagulated whole blood was pipetted and dropped onto the surface of the sample and incubated at 37 °C for 20 min. A total of 1 mL of deionized water was then added to the centrifuge tube along the edge of the tube and the non-coagulated red blood cells were coagulated by shaking and were incubated for 1 min. Subsequently, 0.8 mL of the supernatant was placed into the centrifuge tube and the absorbance of the solution was measured at 545 nm with a microplate reader. The measurement of each sample was repeated three times. Thereafter, 4.55 mL of anticoagulated whole blood was directly added to 1 mL of deionized water and incubated at 37 °C with shaking for 1 min, which served as a positive control. Subsequently, the *BCI* was calculated according to the following formula:

(3) $$BCI\left(\%\right)=\frac{I_{a}}{I_{w}}\times 100\%$$

where $I_{a}$ represents the absorbance of the solution after the recalcified anticoagulated whole blood contacted the sample for a set time and $I_{w}$ represents the absorbance of the solution after the anticoagulated whole blood was mixed with deionized water.

#### 4.4.7. In Vivo Hemostasis Test

A rat liver injury model was used to evaluate the hemostatic ability of the composite hydrogel samples. The rats were randomly divided into three groups (medical gauze, PCZ0, and PCZ3) of three rats in each group. The rats were anesthetized and fixed. After an abdominal incision, the liver was exposed and the fluid around the liver was cleaned. A wound (5 × 5 mm, depth: 3 mm) was created on the anterior lobe of the rat liver using a scalpel. A pre-weighed gauze and samples of the required size (length: 1.5 cm) were immediately administered to the site of the incision on the anterior lobe of the liver with a scalpel. Observations were recorded every 30 s until the bleeding completely stopped to measure the time to hemostasis. Photographs were taken of the wound. Subsequently, the hemostatic materials were weighed to calculate the amount of bleeding after they completely absorbed the blood.

#### 4.4.8. In Vitro Antibacterial Test

A medium preparation of an LB liquid medium was composed of 100 mL of distilled water in a graduated cylinder and poured into a 250 mL reagent bottle. Subsequently, 1 g of tryptone, 0.5 g of yeast powder, and 1 g of sodium chloride were weighed by an analytical electronic balance. The above weighed reagents were added to the reagent bottle and mixed and then sterilized at a high temperature in a high-pressure steam sterilizer at 121 °C for 15 min. An LB solid medium was prepared by measuring 100 mL of distilled water in a graduated cylinder, which was then poured into a 250 mL reagent bottle. A total of 1 g of tryptone, 0.5 g of yeast powder, 1 g of sodium chloride, and 1.5 g of agar powder were weighed by an analytical electronic balance. The above weighed reagents were added to the reagent bottle and mixed and then sterilized at a high temperature in a high-pressure steam sterilizer at 121 °C for 15 min.

For the experiment procedure, PCZ0, PCZ1, PCZ2, and PCZ3 were weighed to 0.1 g, respectively, and sterilized under UV irradiation for 30 min. Before dilution, the concentration of *Escherichia coli* was 1.31 × 10^9^ CFU/mL and the concentration of *Staphylococcus aureus* was 1.01 × 10^9^ CFU/m. The bacterial solution was diluted to 2 × 10^6^ CFU/mL with the LB liquid medium and 10 mL of the diluted bacterial solution was added to a disposable bacterial culture tube. The samples were incubated with *Staphylococcus aureus* (ID: ATCC29213) and *Escherichia coli* (ID: ATCC25922) for 6 h at 37 °C. Thereafter, 10 μL of the suspension was diluted with 1 mL of sterile PBS and 100 mL of the diluted solution was evenly spread onto the solid LB medium. The plates were incubated in an incubator set at 37 °C for 20 h. The plates were imaged. The control was the liquid medium without a sample. The experiment was repeated three times.

#### 4.4.9. Statistical Analysis

All experiments were carried out three times and the data were expressed as a mean ± standard deviation. The results were analyzed with a one-way ANOVA by Origin 2017. A value of *p* < 0.05 was considered to be statistically significant.

## Data Availability

Not applicable.
